# Sex Differences in Appendicitis: A Systematic Review

**DOI:** 10.7759/cureus.60055

**Published:** 2024-05-10

**Authors:** Theofanis F Kollias, Connor P Gallagher, Abdulahad Albaashiki, Venkata Sathya Burle, Ethan Slouha

**Affiliations:** 1 Microbiology, Immunology, and Pharmacology, St. George's University School of Medicine, St. George's, GRD; 2 Medicine, St. George's University School of Medicine, St. George's, GRD

**Keywords:** diagnosing appendicitis, antibiotic therapy, appendectomy, appendicitis, sex differences

## Abstract

Appendicitis is one of the most common gastrointestinal conditions a person can develop. Throughout the years of assessing the different focuses of appendicitis, such as origin, symptoms, labs, diagnosis, treatment, and complications, there have been mere mentions of sex differences. One of the most known sex differences in appendicitis is the fact that males are significantly more likely to develop appendicitis compared to females. Another postulated difference is that males may be more likely to develop a perforated appendix. These differences significantly affect the various aspects of diagnosing and treating appendicitis and may even influence the outcome of appendicitis. Sex difference analysis of conditions has been widely researched over the last two decades, and sex can influence and impact conditions from initial presentation to the outcome of treatment. This paper evaluates the sex differences in appendicitis concerning incidence, risk factors, symptoms, diagnosis technique, treatment, and outcomes across ages. Following PRISMA guidelines, this systematic review reviewed PubMed, ScienceDirect, and ProQuest databases for articles pertaining to sex differences in appendicitis. The original article count was 21,121, which was narrowed down to 28 publications. It was found that, as previously described, males had a significantly higher rate of appendicitis, as well as were at significant risk of perforated appendicitis. No official risk factors were found to differ between the sexes, but males were more likely to complain of symptoms like right lower quadrant cramps/tenderness/pain and loss of appetite. Scores such as the pediatric appendicitis score (PAS) and Ohmann have been used to diagnose appendicitis, but the PAS was significantly more accurate for females, and the Ohmann resulted in significantly fewer negative appendectomies in females as well. Ultrasound and computed tomography (CT) are still the gold standards for diagnosis; however, while time to CT was significantly delayed in females, they were more likely to undergo extensive imaging, possibly to rule out other conditions. Males were more likely to undergo open appendectomies compared to females, who more frequently underwent laparoscopic appendectomy, yet females were more likely to experience complications. Further research should evaluate the influences that can predict postoperative outcomes following appendectomies between sexes and how to prevent/reduce their occurrence.

## Introduction and background

In 1759, Metiever first described appendicitis; however, it wasn’t until the 20th century that the appendix was the actual cause of the condition, leading to the name appendicitis [1]. The appendix is a finger-shaped pouch extending from the cecum as a finger-like projection [1]. Appendicitis results from an inflammation of the appendix due to obstruction, which leads to a backup of the fluids it normally secretes [1]. Various causes lead to the obstruction of the appendix, like infections and inflamed and enlarged lymph nodes [1]. In 2023, the reported incidence of appendicitis was found to be 100 cases per 100,000 person-years in Western nations, and an increasing trend in developing countries [2]. Appendicitis predominantly occurs between 10 and 20 years of age, with an increased risk in males [1]. The lifetime risk of appendicitis is 8.6% in males compared to 6.7% in females [1].

The initial manifestation of appendicitis is peri-umbilical pain from the irritation of the parietal peritoneum that typically shifts to the right lower quadrant (RLQ) of the abdomen, and this is followed in general by fever, nausea, high C-reactive protein (CRP), and increased white blood cell count (WBC) [1,3]. Definitive diagnosis of appendicitis is done via a helical computed tomography (CT) and a Doppler ultrasound (US) with graded compression coloring [1,3]. Previous reports suggest that there is no difference in clinical symptoms like nausea, anorexia, or vomiting between males and females, but there may be some differences in WBCs and imaging results [4]. Various scoring systems assist in making the diagnosis. One of the most frequently used is the Alvarado scoring system, which specifically focuses on the need for surgical intervention [1]. A score between 7 and 10, including symptoms like anorexia, tenderness in the right iliac fossa, rebound tenderness, elevated temperature, leukocytosis, and shift to the left of neutrophils, suggests an emergent need for surgery [1].

Surgery was the original treatment method for appendicitis, precisely an open procedure through making an incision between 1 and 4 inches at McBurney’s point [1]. In 1983, laparoscopic appendectomy was first implemented, allowing for same-day discharge and fewer complications [1]. To this day, laparoscopic appendectomy is the most commonly used treatment for appendicitis [3]. In the mid-1900s, the need for surgery was starting to be questioned as antibiotics may prove to be a safer route with lower morbidity. Still, recurrence rates were high then, indicating the need to save antibiotics for surgically high-risk patients [1]. As of 2021, broad-spectrum antibiotics such as piperacillin-tazobactam alone or with cephalosporins successfully treat uncomplicated appendicitis in the majority of patients [3]. Ultimately, the decision between surgery and conservative management with antibiotics depends on the status of appendicitis, as perforated appendixes require immediate surgery [3]. According to older studies, patients with perforated appendices were also more likely to undergo open appendectomies; males may be more likely to develop perforated appendixes and undergo open appendectomies [4]. While the mortality rate of appendicitis is lower, there have been some reports of an increase in rates as the age of the patient increases and that males may have a slightly higher mortality rate [3].

Aim

Across the period from when appendicitis was first described until now, there have been significant changes in the approaches to diagnosis and treatment of the condition. One aspect not well considered was the influence of sex in appendicitis; however, as of the last two decades, the research in consideration of sex in many diseases has progressed. Few studies throughout this period merely mentioned sex differences noted in secondary outcomes for many conditions. Still, as of this year, more studies have explicitly focused on sex differences. Sex greatly influences how our body reacts to specific pathogens through hormone variations, and there are also sex perceptions when it comes to treatment. This paper aims to assess the sex differences in appendicitis concerning the incidence of acute appendicitis and perforated appendicitis, comorbidities, risk factors, presenting symptoms, diagnostic tests/criteria, treatment, and overall outcome. The goal is to elucidate the differences in treatment and outcomes influenced by the variation in presentation between the sexes and identify possible delays in treatment regarding these sex differences.

## Review

Methods

This systematic review was executed strictly with the Preferred Reporting Items for Systematic Reviews and Meta-Analyses (PRISMA) guidelines presented by Liberati et al. [5]. This involved a focused and methodical search of current literature published in PubMed, ProQuest, and ScienceDirect from January 1, 2003 to December 1, 2023. The keyword "Sex difference in Appendicitis" was used to conduct the search and was chosen to cover the broad spectrum of appendicitis from diagnosis to treatment and treatment outcomes. The initial inquiry across the databases used resulted in 24,121 publications. The investigation only used peer-reviewed observation publications and excluded publications in a language other than English, duplicate publications, and publications prior to 2003. Following the automatic screening process, the selected publications were assessed based on their title, abstract, study type, and full-text availability. A final evaluation of the publication's complete text and ensuring primary and secondary outcomes, including differences or lack of differences in sexes, were present, and the publications were narrowed down to the final count. A total of 28 publications were obtained, and the determination of acceptance was based on the following criteria.

Inclusion Criteria

The selected publications consisted of the following criteria: studies evaluating or reporting sex variations in appendicitis, peer-reviewed experimental or observational studies, full-text availability, publications following 2003, and studies performed on humans.

Exclusion Criteria

**Figure 1:**
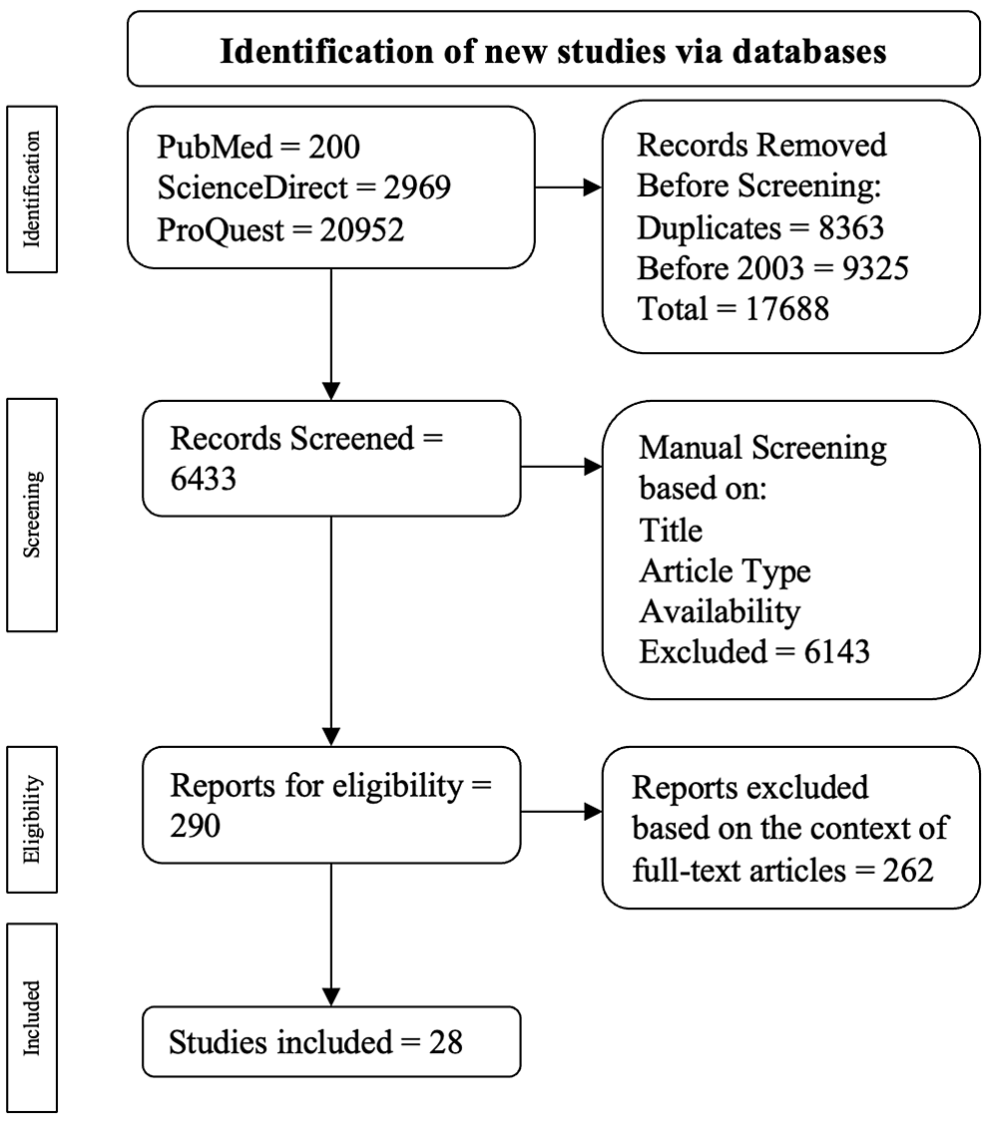
Algorithm employed using based on the inclusion and exclusion criteria The flowchart was adapted to PRISMA guidelines [5]. PRISMA: Preferred Reporting Items for Systematic Reviews and Meta-Analyses

Excluded publications were based on the following criteria: publication in a language other than English, no full-text availability, case reports/series, meta-analysis, systematic reviews, and duplicate publications. The screening process using the mentioned inclusion and exclusion criteria is drawn out in Figure 1.

Bias

All publications acquired were evaluated for bias through the GRADE (grading of recommendation, development, and evaluation) scale with the specific observational cohort scale. With ample sample sizes, detailed procedures, and thoroughly explained statistical analysis in all studies, a low-moderate bias rating was given.

Results

About 24,121 publications were populated in the initial search of which 200 were from PubMed, 2969 were from ScienceDirect, and 20952 were from ProQuest. Among the exclusions, 8363 were duplicate publications, and 9325 were from before 2003. This resulted in 17688 publications being excluded throughout the automatic inspection, leading to 6433 publications for manual screening. Publications were then evaluated centered on their title, full-text availability, and study type, resulting in 290 publications selected for full-text examination eligibility. Ultimately, 28 publications were chosen. The articles used to compile this review are seen in Table 1.

**Table 1:** Summaries of the articles used in this review per PRISMA Guidelines [5] CT: computed tomography; US: ultrasound; LA: laparsocopic appendectomy; PA: perforated appendicitis; NPA: non-perforated appendicitis; LOS: length of stay; WBC: white blood cells; CRP: C-reactive protein; AAp/AA: acute appendicitis; NA: negative appendicitis; MD: missed diagnosis; SED: single encounter diagnosis; NFHCT: non-focused helical CT; RLQ: right lower quadrant

	Author	Country	Design and study population	Findings	Conclusion
1	Abdelhalim et al., 2015 [6]	United Kingdom	Retrospective Cohort Study (n = 363)	There was a significantly higher prevalence of males at 62% compared to females at 38%. 53% of female patients received pre-diagnostic imaging compared to 28% of male patients. There was a difference in the sexes in that CT scans were utilized by 72% of males compared to 40% of females, of whom the US was the favored imaging modality. Finally, there was a difference between the sexes in that LA was favored in females and attempted first in 72% of females compared to males accounting for 31%.	There was a difference between the chosen imaging modality between sexes in that males received more CT scans than females, and females underwent US more than males.
2	Akbulut et al., 2021 [7]	Turkey	Retrospective Cohort Study (n = 1,316)	The study identified male sex, WBC ≥ 10,900, and CRP ≥ 0.725 as independent risk factors for developing acute appendicitis. The most prevalent age group was localized to the second and third decade of life for developing AAp. However, developing perforation was localized to the fifth decade.	Male sex is a risk factor for developing acute appendicitis, although sex was not a contributing risk factor for perforation.
3	Augustin et al., 2011 [8]	USA	Retrospective Cohort Study (n = 380)	Males have an increased prevalence of perforated appendicitis than females. There was also evidence suggesting that males have an increased risk of perforation in the first 36 hours of diagnosis compared to females. Males were found to have a shorter duration of perforating their appendix when compared to females.	There was a suggested finding that there is a risk of early perforation with a diagnosis of acute appendicitis. In this finding, it was expressed that males above 55 are at highest risk for early perforation.
4	Avci and Ayengin, 2019 [9]	Turkey	Retrospective Cohort Study (n = 267)	The incidence of acute appendicitis was higher in males, accounting for 63%. However, the number of perforated appendices was higher in females at 59% when compared to males and associated with a longer LOS.	The rate of perforated appendix was significantly higher in females than males, despite males having a significantly higher incidence of appendicitis
5	Bachur et al., 2012 [10]	USA	Retrospective Cohort Study (n = 55,227)	In male children, the rate of NA was 16.8%, significantly higher than in females, which was 14.6% for patients below the age of 5. However, after the age of 5, females were significantly more likely to have NA	This study suggests that there is a difference in negative appendectomy rate based on imaging modality when comparing both age and sex.
6	Barışık et al., 2020 [11]	Turkey	Prospective Cohort Study (n = 643)	There is a statistical significance between the age of appendicitis diagnosis in males and females, as well as BMI, cigarette smoking, sheesha smoking, family history of diabetes, and hypertension. There was also statistical significance in the perception of pain of acute appendicitis between males and females.	CT was more cost-effective than the US when diagnosing appendicitis. This study also identified potential factors as being high risk, some of which are nausea, vomiting, guarding, and loss of appetite.
7	Gil et al., 2023 [12]	USA	Prospective Cohort Study (n = 51,164)	A significantly lower proportion of those diagnosed with appendicitis were females. 98.2% of the population had SED, and 1.8% had MD, the strongest predictor of appendiceal perforation. MD’s were more likely to occur in females.	Minority race and females at high risk of MD and appendiceal perforation. The high rate of MD correlates to errors in clinical judgment and the decision not to pursue additional differential tests.
8	Günay et al., 2022 [13]	Turkey	Case-Control Study (n = 186)	Marked increases in Rho-kinase 1 and mRNA expression were observed in male patients. No significant variation was observed in alleles and genotype frequencies for Rho-kinase 1 genes rs35996865 and Rhino-kinase genes rs2230774 polymorphisms in AA patients and the control group.	Rho-kinase 1 and Rho-kinase 2 genes were no significant AA development risk factors. However, increased expression of mRNA of the Rho-kinase 1 genes in males suggested that this gene predicts the pathogenesis of AA in a sex-specific manner.
9	Kambouri et al., 2019 [14]	Greece	Retrospective Cohort Study (n = 602)	AA diagnosis for males was delayed for at least 48 hours in 47.7%. Males diagnosed but did not take antibiotics had lower odds of a prolonged diagnostic period greater than 48 hours. Females who took antibiotics are 12 times more likely than those who do not take antibiotics to have a longer diagnostic period greater than 48 hours.	Physicians assessing children with abdominal pain should consider various causes of diagnostic delay that can contribute to serious complications and prolonged hospital stays.
10	Lin et al., 2015 [15]	Taiwan	Population-Based Study (n = 294,544)	The incidence of appendicitis was 107.76 per 100,000 person-year, with the highest incidence between 15-29 years of age. Males had a significantly higher rate than females, except those over 70. Males with appendicitis were more likely to have a perforated appendix.	Males had a higher incidence of appendicitis and were more likely to develop perforated appendicitis compared to females.
11	Salö et al., 2015 [16]	Sweden	Retrospective Cohort Study (n = 427)	The rates of phlegmonous appendicitis, perforated appendicitis, open appendectomy, and RLQ tenderness with percussion/coughing/hopping were higher in males. In females, negative appendectomy, operative complications, gangrenous appendicitis, and pre-operative imaging were found to be higher.	Sex differences were noted, with males having appendicitis with perforation more than females and females had higher rates of negative appendectomies and operative complications.
12	Seow et al., 2022 [17]	Singapore	Retrospective Cohort Study (n = 1,185)	Males represented 79.3% of the sample, significantly more than females. The average duration of symptoms was 1.8 days with a mean LOS of 3.6 days, with males being a risk factor for prolonged LOS	Males were significantly more likely to develop appendicitis and were a risk factor for a prolonged LOS
13	Weiss et al., 2023 [18]	Israel	Retrospective Cohort Study (n = 1,839)	Males were found to have a significantly lower chance of receiving analgesia; however, males were associated with a significantly higher chance of receiving a minimum of one opioid medication, assuming that they were started on pain medication.	Males were not as likely as females to receive analgesics for pain management, but when given analgesics, males were more likely to receive at least one opioid.
14	Zvizdic et al., 2021 [19]	Bosnia	Retrospective Cohort Study (n = 295)	31.2% of patients had a perforated appendix. The male sex, fever, diarrhea, vomiting, high CRP levels, and elevated WBC levels were found to be predictors of perforated appendicitis.	Predicting factors to help distinguish between perforated and non-perforated appendicitis are the male sex, fever, CRP levels, and elevated WBC levels.
15	Ahmed et al., 2020 [20]	Iraq	Prospective Case Series Study (n = 5,847)	This study found that the most likely age group for acute appendicitis was between the age ranges of 21-40; within this age range, the female-to-male ratio was 1.49:1.00, respectively. This study found that the most likely age group for suspected appendicitis was the age range of 31-40, which had a female-to-male ratio of 1.5:1.00 respectively. The most common cause of morbidity in both sexes was surgical site infection.	Female patients have more postoperative complications than males when receiving appendectomy. However, no statistically significant findings suggest an increase between sexes in the development of appendicitis.
16	Şenocak et al., 2020 [21]	Turkey	Retrospective Cross-Sectional Study (n = 202)	For both sexes, the Ohmann scoring system was found to identify acute appendicitis the best, resulting in the lowest negative appendectomy rates (3.4% in males and 6.9% in females). Although combining the scores with ultrasonography did not increase the accuracy of diagnosing acute appendicitis, combining the Ohmann scoring system with ultrasonography resulted in a decrease of negative appendectomy rates for females from 6.9% to 4%.	The Ohmann score provided the best negative appendectomy rate and is a good diagnostic predictor of acute appendicitis in both male and female patients. When combined with ultrasonography, it resulted in a further decrease in the female negative appendectomy rate while minimally reducing the rate for females.
17	Drake et al., 2014 [22]	USA	Prospective Cohort Study (n = 9,048)	There was a significantly higher amount of males (55.3%) who developed a perforated appendix compared to females (52.1%), and males were also found to be an independent risk factor for developing PA. Males also had a shorter time to treatment following diagnosis compared to females.	Perforation was correlated with the male sex, who also had a significantly shorter time frame from diagnosis to treatment.
18	McGann Donlan and Mycyk 2009 [23]	USA	Retrospective Cohort Study (n = 137)	The time from triage to CT order was 95 minutes in males and 138 minutes in females. The time from initial physician evaluation to CT order was 28 minutes in males and 45 minutes in females. Nonclassic symptoms were prevalent in females, and pelvic evaluation did not delay the CT order.	Female sex is associated with delays in CT acquisition and diagnosis of appendicitis.
19	Stein et al., 2012 [24]	Israel	Retrospective Cohort Study (n = 3,736)	Males were found to have more appendicitis attacks, while females were found to have a greater number of normal appendixes. The summer season months had increased numbers of appendectomies for acute appendicitis. There was a 0.33% mortality rate, with the majority being elderly and female, as the male:female ratio was 0.4.	Acute appendicitis was found to be more frequent in the summer season and the male sex. Females also had a slightly higher mortality rate than males.
20	Dohner et al., 2023 [25]	Switzerland	Retrospective Cohort Study (n = 457)	There were slightly more males diagnosed with appendicitis during this time frame compared to females, but neurogenic appendicopathy was diagnosed more frequently in females. Female sex was associated with more frequent in these patients than in those with acute appendicitis.	While males were more likely to be diagnosed with appendicitis, females were more likely to be diagnosed with acute and neurogenic appendicopathy.
21	Lien et al., 2011 [26]	Taiwan	Prospective Cohort Study (n = 128)	There was no significant difference in NPA and PA groups with recurrence rate; however, male sex was associated significantly with recurrence.	There was a significant risk of recurrence of appendicitis in males.
22	Oguntola et al., 2010 [27]	Nigeria	Retrospective Cohort Study (n = 299)	52% of patients in this study were males. Admission rates of appendicitis in both sexes have increased since 2004. The incidence of appendicitis in both sexes was significantly higher during the rainy season.	There is an increase in the incidence of appendicitis in males, but the overall incidence in both sexes has been increasing since 2004.
23	Sulu et al., 2010 [28]	Turkey	Retrospective Cohort Study (n = 1,871)	Appendicitis was seen mostly in males between the ages of 10 and 19, while perforate appendicitis was seen the most in the 0-9 and 50+ years age groups. Appendicitis was seen mostly in the winter months, while perforation rates were the lowest.	Appendicitis frequency was affected by age, sex, and possibly by seasons and temperatures.
24	Livingston et al., 2007 [29]	USA	Retrospective Cohort Study	Between 1970 and 1995, there was a decrease in the negative appendectomy rate in females. Nonperforated rates have been oscillatory, with large peaks in the male population. The rate of perforated appendicitis and abscesses has decreased in both sexes.	There’s been a decline in non-perforated appendicitis and an increase in appendectomies associated with the implementation of new technology
25	Peeters et al., 2023 [30]	Belgium	Retrospective Cohort Study (n = 9,723)	Male sex was found to be a significant independent risk factor in developing appendicitis.	Males are at an increased risk of developing appendicitis
26	Raman et al., 2003 [31]	USA	Retrospective Cohort Study (n = 552)	The sensitivity of NFHCT to diagnose appendicitis was significantly higher in males, while specificity was slightly higher in females. Accuracy of the diagnosis, however, did not differ between sexes. Males were slightly more likely to have thickened appendix and periappendiceal stranding.	NFHCT may be highly accurate in diagnosing appendicitis.
27	Peixoto et al., 2011 [32]	Brazil	Prospective Cohort Study (n = 156)	Sex differences did not alter the diagnostic accuracy of abdominal ultrasonography to diagnose appendicitis.	Sex does not influence the accuracy of ultrasound to diagnose appendicitis.
28	Akbulut et al., 2019 [33]	Turkey	Retrospective Cohort Study (n = 360)	No statistically significant data was identified that suggested a link between the sexes of patients and their likelihood of receiving a diagnosis of acute appendicitis. There were also no differences between the sexes regarding the incidence of incidental and emergent appendectomies.	The study did not find any significant link/difference with regards to sex linking patients who received incidental appendectomy and those who received emergent appendectomy.

Males tended to have a significantly higher prevalence and incidence of appendicitis compared to females, with the most prominent peak of appendicitis in males between 15 and 19 years of age [6-19]. It was also observed that males were significantly more likely to develop a perforated appendix compared to females, who were more likely to develop neurogenic appendicitis [12,20,21]. Seasonal trends were evaluated in one study, and it was found that there was an increase in appendicitis diagnoses in the summertime for both males and females [15]. Common risk factors for appendicitis were advanced age and male sex, with risk factors for a perforated appendix being male sex, duration of symptoms, and elevated WBC count [13,17,22]. When it comes to symptom presentation, males were more likely to have RLQ pain/cramps/tenderness and anorexia [11,16,23]. Still, there were conflicting results on sex differences in nausea, vomiting, and diarrhea. Patients with perforated appendicitis tended to have significant fevers, vomiting, high WBC concentrations, and high CRP concentrations.

US and CT are standard imaging techniques for diagnosing appendicitis, and it was found that while females experienced a significant delay in receiving these scans, they were more likely to go through more imaging than males [6,10,16]. The Pediatric Appendicitis Score (PAS) used in pediatric patients, which classifies appendicitis, was found to be significantly more accurate in diagnosing appendicitis for females than for males under 18 [16]. Treatment of appendicitis can be done through conservative management with antibiotics or surgery. However, no study went into depth on antibiotic regimens. Males were more likely to undergo open appendectomies with longer surgical times than females undergoing open appendectomies. In contrast, females were more likely to undergo laparoscopic appendectomies, also experiencing longer surgical times compared to males undergoing laparoscopic appendectomies. Females overall were found to experience higher rates of complications during the treatment of appendicitis, but in both sexes, wound infections were the most common complication [6,7,16,24]. While the mortality rate is low, there were inconclusive reports on whether or not mortality was increased in either sex. Last, one study evaluated the cost of appendicitis between the sexes, and it was found that males suffering from any form of appendicitis (acute/perforated) cost more than females [15].

Discussion

Incidence of Appendicitis Between Sexes

Appendicitis can occur in any individual, with one report indicating 107.76 per 100,000 person-years [15]. Across all studies examined, the average age at appendicitis diagnosis was 34.6, and one study stated that the median age was 43.2 [6,13,17,22-28]. Two studies also evaluated seasonal pattern variations in the diagnosis of appendicitis. However, they had conflicting observations, with one indicating that appendicitis was higher in the spring and the other in the winter [27,28]. Trends of appendicitis throughout the years also have varying reports. Oguntola et al. found that the incidence of appendicitis is upward for both sexes [27]. Meanwhile, Lin et al. determined that a downward trend was occurring in both sexes [15]. Despite the improvement in diagnostic testing, it is also reported that the incidence of perforated appendicitis is increasing in both sexes, with one study reporting up to 31.2% [19,29].

While appendicitis occurs in both sexes, studies found that the incidences between males and females differ. The average incidence of males across studies was 54.7%, with one study reporting an incidence of 114.38 per 100,000 person-years and an average age of 36.3 years [6-20,22-28,30,31]. The most prominent peak of appendicitis in males was seen in 15-19 years old, with an incidence of 152.92 per 100,000 person-years [15]. Lin et al. also observed seasonal variations of appendicitis in males and found that during the summer, the incidence of appendicitis was significantly higher by 11.76% [15]. Perforation of the appendix is a complication of appendicitis, and about 20.24% of males develop this complication [7,8,15,22,28]. One study evaluated that the male children had a 3.13 greater odds of having a perforated appendix vs. a non-perforated appendix [19]. The reported incidence of perforated appendix in males is 31.59 per 100,000 person-years [15].

However, the incidence of appendicitis in females was an average of 39.3% and 100.96 per 100,000 person-years in one study [6,7,10-20,22-24,27,28,30,31]. The mean age at diagnosis of appendicitis was 35.9 years across studies that reported age, and peak occurrence was 20-24 years old [23,27,28,30,31]. Lin et al. evaluated season changes as well in females diagnosed with appendicitis and observed that there was a significant increase of 14.56% in diagnoses during summertime [15]. However, an average of 33.75% of females with appendicitis developed a perforated appendix, with Lin et al. reporting an incidence of 22.69 per 100,000 person-years [8,15,22,28,31]. Another complication of appendicitis is the diagnosis of neurogenic appendicitis, which occurred in 65% of females in one study [25]. Another common problem associated with appendicitis is a missed diagnosis, which occurred in females at 47.9%; the same study also showed a 1.45 increase in the odds of having a missed diagnosis for females [12].

Comparing the sexes, the majority of studies observed that there was a significantly higher incidence of males being diagnosed with appendicitis [6-19,26]. However, a few studies observed the opposite, with females having a higher incidence of appendicitis diagnosis [20,30,31]. One study observed that females tended to be significantly younger than males at diagnosis and that males had a higher incidence for all age groups except >70 [15,25]. Several studies observed that males were significantly more affected by acute appendicitis, with male-to-female ratios ranging from 2.6:1 to 1:0.03 and a rate of 1.5 times more [9,13,20,21,24,27].

Complications like a perforated appendix also occurred significantly more in males than females at 63.5% and a ratio of 1.39:1 males to females; however, some studies specifically covering the incidence of perforation found different percentages with 20.24% of males compared to 33.75% of females developing perforation [7,8,15,19,22,28,31]. However, Avci and Ayengin observed that perforated appendicitis was more likely to occur in females, which may be due to a later presentation to the emergency department [9]. Missed diagnosis, when it does occur, is more likely to happen to females as well; at the same time, females are at a higher risk of undergoing a negative appendectomy [12,20,21]. Sexes did share comorbidities such as hypertension, asthma, ischemic heart disease, malignancy, diabetes, and hyperlipidemia, but no study reported differences in comorbidities [20,24]. With these results, Avci and Ayengin suggest that sex plays a component in the development of the pathology, but Augustin et al. contrasted this, stating there was no influence occurring [8,9].

Differences in Risk Factors of Appendicitis Between Sexes

Most studies observed that being male was a factor in developing appendicitis [7,14,22,30]. McGann Donlan and Mycyk, however, reported that being female was a significant risk factor as the incidence of females with appendicitis was significantly higher in their study [23]. No studies reported the difference in risk factors between sexes in developing appendicitis, but males have an estimated lifetime risk of 8.6% of developing acute appendicitis, while females have 6.7% [7]. Common risk factors of acute appendicitis are advancing age, three or more comorbid conditions, and, according to one study, an increase in ROCK1 gene expression [13,22]. In complicated appendicitis, male sex was deemed a significant risk factor, along with age, duration of symptoms, race, laparoscopic approach, race, elevated WBC count, low-income individuals, medical center use, regional hospital use, diarrhea, and fever [17]. However, in the pediatric population, it was noted that the female sex was associated with perforation and not males [12]. Contrary to most studies, Akbulut et al. reported no apparent correlation between sex and the risk of a perforated appendix [7].

Differences in Presenting Symptoms Between Sexes

The most common symptoms of appendicitis were fever, nausea, vomiting, anorexia, diarrhea, dysuria, and abdominal pain with tenderness in the RLQ on physical exam [17,19]. Males specifically were significantly more likely to have symptoms such as RLQ pain, cramps, tenderness, fever, weakness, bloating, loss of appetite, anorexia, constipation, positive bowel sounds, nausea, and vomiting [11,16,23]. However, two studies observed that females were significantly more likely to have nausea and vomiting, along with diarrhea [16,23]. Dysuria rigidity, obturator sign, psoas sign, and Rovsing’s did not differ significantly between sexes [11]. Specific symptoms associated with perforated appendicitis were fever, vomiting, elevated WBC concentration, diarrhea, and high CRP [19]. Zvizdic et al. observed symptoms in children and determined that if the child was vomiting, had a fever, or had diarrhea, they had 2.34, 3.43, and 4.59 times greater odds of having a perforated appendix compared to non-perforated [19].

Difference in Tests to Diagnose Appendicitis Between Sexes

Appendicitis leads to alterations in the biochemical survey of patients, such as increased WBC and CRP, as well as physical changes on imaging. One study observed that leukocytosis, CRP values, and neutrophilia rates were not significantly different between the sexes in the pediatric population [16]. In children, initial scores like the PAS were used to establish/rule for appendicitis prior to imaging [16]. The PAS was found to identify appendicitis in females more frequently at 59% than in males at 41% [16]. With this, it was observed that the PAS was significantly more accurate when used in females than for males at 33% and 11%, respectively [16]. A new diagnostic tool, the Ohmann tool, has been trialed [21]. Ohmann was found to be the most predictive tool for diagnosing acute appendicitis for both sexes >18 years of age, with the lowest negative appendectomy rates in females, especially when combined with US [21]. However, in combination with the US, the Ohmann tool did not increase the accuracy rate of diagnosis of acute appendicitis [21]. Another method used to diagnose appendicitis was the Alvarado and modified Alvarado scores, but no differences in accuracy between the sexes were observed [22].

Imaging is the gold standard for a definitive diagnosis of appendicitis, CT specifically, and it was found that females of all ages were more likely to receive further pre-operative imaging than males overall [6,10,16]. It was proposed that this could be related to the relative uncertainty in diagnosing appendicitis with right iliac fossa pain as the main associated symptoms [6]. In contrast to most studies, two studies observed a difference in use, with Salö et al. stating similar use between sexes and Abdelhalim et al. reporting that CT was utilized significantly more in males [6,16]. However, McGann Donlan and Mycyk observed delays in ordering CT scans in both males and females [23]. Raman et al. assessed the efficacy of non-focused helical CT (NHCT) and found that the overall accuracy did not differ between sexes. However, NHCT had significantly higher sensitivity in males and significantly higher specificity in females [31]. The imaging showed that males were slightly more likely to have a thickened appendix and periappendiceal fat stranding [31]. With the US as pre-operative imaging, there was no difference between the sexes. However, Peixoto et al. observed a slightly better accuracy in diagnosing male appendicitis [16,32].

Treatment

Regarding treatment, it was essential to determine the differences between the sexes, considering emergency department presentation, antibiotic vs. surgery, surgery type, and pain management following surgery. Seow et al. observed that the overall average duration of symptoms prior to presenting to the emergency department was 1.8 days [17]. Studies that reported this data mostly stated no difference in time to the emergency department between the sexes [8,16]. However, one study observed that females took longer to come to the emergency department following the onset of symptoms at 3.39 days compared to 2.21 days for males [9]. Regarding the perforation of the appendix, whether or not presenting to the emergency department, males had shorter symptom duration than females [8]. Once in the hospital, the previous two studies also observed no difference between the initial triage of symptoms and the diagnosis of appendicitis between the sexes [8,16]. Still, Drake et al. observed that males received a shorter treatment time of 8.2 hours, while females received treatment in 9 hours [22].

Initial treatment of appendicitis typically involves antibiotics, either for prophylaxis before surgery or as an actual treatment of appendicitis. However, none of the studies found referenced the use of antibiotics as the primary treatment for appendicitis. Lien et al., however, did mention that males accounted for 53% of non-surgical patients but did not go further into the alternative treatment [26]. Typically, though, antibiotics were administered within 24 hours of diagnosis prior to surgery [14,25], the average duration of antibiotic use from diagnosis to post-operation was 21 days. Regarding complications of appendicitis, such as gangrene or perforation, there was no significant difference in antibiotic duration between sexes [15,16].

As surgery was the first choice for the studies in this review, the decision for surgery was determined by a pre-operative CT scan of the abdomen and pelvis in 42.9% of patients [17]. In another study, surgery was decided for 57.1% of patients in addition to CT scans based on CRP levels, neutrophil-WBC ratio, and clinical symptoms [17]. Ultimately, acute appendicitis was confirmed during surgery in 84.2% of Dohner et al.’s study [25]. The incidence of primary appendectomies in males was 107.83 per 100,000 person-years, whereas in females, it was 95.15 per 100,000 person-years with a male:female ratio of 1.13:1 [15]. This is compared to incidental appendectomies, which were up to five times more common in females despite being on the decline in both sexes [29]. Akbulut et al. observed that there was, however, no difference regarding sexes and the incidence of incidental and emergency appendectomies [33].

Surgery for appendicitis either consists of an open or laparoscopic approach. Over the years, the rate of open appendectomies has declined while laparoscopic approaches have increased [29]. Across several studies, the average prevalence of open appendectomies was 73.57%, with males having significantly higher rates of undergoing open appendectomies [6,7,16,24]. However, compared to females, males who underwent open appendectomies had significantly longer operation times. In one study, all patients underwent laparoscopic appendectomy, but excluding this study, the average prevalence of laparoscopic appendectomies across the remaining studies was 40.48%, with females being significantly more likely to undergo laparoscopic appendectomy [6,7,13,17,24]. At the same time, compared to males, females who underwent laparoscopic appendectomies had significantly longer operation times [15,16].

Post-operation, patients are also given various analgesics per physician preference and the severity of pain experienced. Weiss et al. reported that 48% of patients did not receive analgesia, 31% received non-opioid analgesia, and 21% received at least one dose of opioids. Patients who were likely to receive at least one dose of opioids typically fell in the age range of 25-64 years old. Salö et al. observed no significant difference between the sexes regarding administering any form of analgesia post-operation. However, males had a significantly lower chance of receiving any form of analgesic medications, but of those who did receive analgesia, males had a significantly higher chance of being given a minimum of one dose of opioids [16,18].

Outcome

Following the diagnosis of appendicitis, the duration of the hospital stay varied between the types of appendicitis as well as whether or not pharmacological treatment was used over surgery. Across studies, the overall duration of hospital stay for appendicitis was 3.51 days [13,15,17,28]. Compared to non-perforated appendicitis, perforated appendicitis required a significantly longer hospital stay duration, with one study reporting up to 7.55 days on average [15,28]. Analgesia use following surgery also altered the hospital stay, according to Weiss et al., who report that patients who were given analgesia had longer hospital stays [18]. Two studies reported that males had significantly longer durations of hospital stay compared to females in both non- and perforated appendicitis with a ratio of 1.01-1.05:1. They were also found to be a significant independent risk factor for a prolonged length of stay [15,17]. However, Salö et al. found no difference between the sexes in the time spent at the hospital [16]. Postoperative patients’ median follow-up period after discharge ranged from 12 to 40 months [16,26]. Following antibiotic use, whether for treatment or prophylaxis/following surgery, diarrhea was the most common complication and was reported as tolerable [26]. About 16% of patients who were managed through medications developed recurrent appendicitis, with a significant proportion being males [26]. Of those recurred, 50% resulted in a perforated appendix [26]. The 30-day readmission rate was observed in 2.4% of patients, according to Seow et al. [17]. Males had a significantly higher readmission rate at 3.89% compared to females at 2.27% [15].

Focusing on complications following surgery, the overall incidence of morbidity was low, with an average of 6.2% [13,17,20,23,25]. Two studies reported no complications during hospitalization [13,23]. Common complications observed following post-operation were wound infection at the surgical site, ileus, intestinal obstruction, and septicemia [17,20]. Of those patients with complications, 1.6% had serious complications resulting in critical care through an integrated team [17]. Two studies evaluated the rates of wound infections, and all three found different results, either with males more likely to experience wound infection or females experiencing more wound infections; however, it is agreed that wound infection was the most common complication between the sexes [20,24]. Compared to females, males had a higher incidence of peritonitis and incisional hernias [24]. Overall, females were found to have higher rates of complications following the operation, with higher incidences of abscess and multi-organ failure [16,20,24]. However, Salö et al. observed these differences as insignificant, resulting in no difference between postoperative complications or need for operation between the sexes [16].

The mortality rate following appendicitis is also significantly low, with an average of 0.43% in studies reporting mortality [17,20,23,24]. Ahmed et al. observed that 1.23% of their sample deteriorated and died within the first month following their appendectomy [20]. Stein et al. observed a big decrease in the mortality rate of true appendicitis from the winter to spring, summer, and autumn [24]. Only two studies evaluated the differences in mortality when comparing the sexes. Lin et al. found that the case-fatality ratio was higher in males at 0.14% compared to 0.09% in females, but this was insignificant [15]. Whereas Ahmed et al. found that the mortality rate was non-significantly higher in females at 0.49% compared to 0.29% in males [20].

Cost

Only one study evaluated the cost of appendicitis between the sexes, which may further elucidate the differences in treatment and outcomes as this is where the expenses lie. The total cost of appendicitis overall was $1,042, with acute costs of $1,029, primary costs of $1,104, and perforated costs of $1,457. They found that the cost of treating appendicitis, acute appendicitis, and perforated appendicitis was higher in males than in females by 2.13%, 2.26%, and 0.9%, respectively. Whereas for primary appendicitis, treatment for females is higher at 2.59% [15].

Limitations to this study cross between the sections focused on in this paper. Not enough studies focused on the sex differences in risk factors of appendicitis, which should be focused on as this can allow for a tailored management and treatment regimen. This regimen would also be influenced by studies focusing more on the differences in treatment between the sexes and identifying if there’s an optimal treatment or a bias in treatment concerning the sexes. Currently, the studies found only highlight open vs. laparoscopic surgery, but recent studies on appendicitis treatment indicate that medical management is becoming the mainstay. One of the strengths this paper offers is a comprehensive analysis of the outcomes of appendicitis between the sexes, as this will allow physicians to identify potential complications and be ready to address them when they occur.

## Conclusions

Throughout the years, various aspects of appendicitis have been analyzed, such as the origin of the condition, the symptoms associated with the condition, risk factors associated with the condition, the treatment of the condition, and complications following the treatment. Throughout this time, the incidence of appendicitis has increased, and the need for further evaluation has come to light as previous studies have mentioned some differences between sexes concerning appendicitis. It is well known that males have a higher incidence of appendicitis, and it has been mentioned that males may be more likely to experience perforated appendixes. This review showed that incidence and complications are different in all stages of appendicitis, from diagnosis to outcomes of treatment. Morbidities are still present following either form of treatment mentioned, and differences between the sexes are also shown. As appendicitis is a very common condition, there needs to be a focus on the varying aspects that could also try to reduce the incidence. One of the ways this can be achieved is by extensively looking at and comparing variations in sex and answering questions like “Why are males more likely to develop appendicitis?” Modern medicine is constantly evolving to improve the care of patients for any condition, and appendicitis should not be left out because of the significantly low mortality.
